# Supplementary material for: Generation of a Realistic Synthetic Laryngeal Cancer Cohort for AI Applications
Source: Cancers (Basel). 2024 Feb 1;16(3):639. doi: 10.3390/cancers16030639 (PMC10854797; doi:10.3390/cancers16030639)
Supplement: Supplementary file 1 [file cancers-16-00639-s001.zip › cancers-2775315-supplementary.pdf]

**Table S1.** Comparison of synthesized data (cohort approach, data set C32-3) with published data from cancer registries or literature. If more than one source was used, the median value and the range (in brackets) are displayed.

| Variable | | Synthea data | | Validation data | | Validation source |
|---|---|---|---|---|---|---|
| | | **female** | **male** | **female** | **male** | |
| gender (%) | | 14.7 | 85.3 | 13.3 | 86.7 | ZFKD, 1999-2019 [31] |
| age group (%) | 40 - 49 | 8.7 | 6.1 | 8.3 | 6.6 | ZFKD, 1999-2019 [31] |
| | 50 - 59 | 24.0 | 23.7 | 24.9 | 24.2 | |
| | 60 - 69 | 32.9 | 35.1 | 31.4 | 34.5 | |
| | 70 - 79 | 22.1 | 25.0 | 23.9 | 25.8 | |
| | > 80 | 12.3 | 10.1 | 11.5 | 9.0 | |
| T-stage (%) | T1 | 36.4 | 43.2 | 35.9 (29.5 - 42.1) | 45.3 (44.3 - 46.2) | CR Baden-Württemberg [32], CR Niedersachsen [33] |
| | T2 | 23.5 | 21.0 | 25.3 (21.0 - 29.5) | 19.3 (19.0 - 19.5) | |
| | T3 | 23.9 | 18.9 | 23.2 (22.7 - 23.7) | 19.8 (19.0 - 20.5) | |
| | T4 | 16.2 | 16.9 | 15.7 (13.2 - 18.2) | 15.7 (13.8 - 17.6) | |
| N-stage (%) | N- (N0) | 67.3 | 73.9 | 63.0 (58.1 - 69.0) | 78.2 (77.0 - 79.4) | CR Baden-Württemberg [32], CR Niedersachsen [33], Stage Migration and Survival Trends in Laryngeal Cancer [34] |
| | N+ | 32.7 | 26.1 | 35.5 (31.0 - 41.9) | 21.8 (20.6 - 23.0) | |
| | N1 | 7.9 | 7.0 | 8 | | |
| | N2 | 22.0 | 16.5 | 21 | | |
| | N3 | 2.8 | 2.6 | 2 | | |
| M-stage (%) | M0 | 95.5 | | 98.0 (96.9 - 99.0) | | CR Baden-Württemberg [32], CR Niedersachsen [33], CR Schleswig-Holstein [35], Laryngeal cancer: epidemiological data from Northern Greece and review of the literature [36] |
| | M1 | 4.5 | | 2.1 (1.0 - 3.1) | | |
| UICC Stage (%) | I | 34.9 | 40.5 | 27 | 37 | KID, 2021 [18] |
| | II | 15.4 | 14.5 | 14 | 14 | |
| | III | 15.1 | 15.1 | 22 | 17 | |
| | IV | 34.6 | 29.9 | 37 | 33 | |
| Localization (%) | Supraglottis | 20.0 | | 31.1 (27.2 - 34.9) | | CR Baden-Württemberg [32], CR Niedersachsen [33], Laryngeal cancer: epidemiological data from Northern Greece and review of the literature [36] |
| | Glottis | 77.1 | | 66.9 (64.0 - 69.8) | | |
| | Subglottis | 2.9 | | 2.8 (1.1 - 4.4) | | |
| Grading (%) | G1 | 6.9 | | 10.3 (8.6 - 11.9) | | CR Niedersachsen [33], CR Schleswig-Holstein [35] |
| | G2 | 68.9 | | 69.3 (67.0 - 71.7) | | |
| | G3 | 24.2 | | 20.4 (16.4 - 24.4) | | |
| Smoker | non-smoker | 12.0 | | 5.6 | | Laryngeal cancer: epidemiological data from Northern Greece and review of the literature [36] |
| | smoker | 46.2 | | 86.9 | | |
| | former smoker | 41.8 | | 7.6 | | |

**Table S2.** Comparison of synthesized 1- and 5-year survival with the Cancer Registry Data set Schleswig-Holstein (CR SH) and external reference data. The star (*) indicates a significant difference between Synthea and CR SH.

| | Synthea | CR SH | External reference data | Source |
|---|---|---|---|---|
| Survival by gender 1-year | m: 86.5%<br>f: 85.3% | m: 87.6%<br>f: 86.6% | m: 86.7%<br>f: 85.9% | Cancer registry Munich [35] |
| 5-years | m: 58.1%<br>f: 57% | m: 61.0%<br>f: 63.9% | m: 60.3%<br>f: 60.1% | Cancer registry Munich [35] |
| Survival by age-groups 1-year | <50: 91.1%<br>50-59: 87.9%<br>60-69: 88.5%<br>>70: 82.1% | <50: 90.0%<br>50-59: 89.9%<br>60-69: 89.3%<br>>70: 82.8% | <50: 93.6%<br>50-59: 90.6%<br>60-69: 86.4%<br>>70: 80.9% | Cancer registry Munich [35] |
| 5-years | <50: 68.8%<br>50-59: 68.4%<br>60-69: 61.1%<br>>70: 44.8% | <50: 68.0%<br>50-59: 70.6%<br>60-69: 63.6%<br>>70: 49.3% | <50: 73.3%<br>50-59: 67.7%<br>60-69: 60.1%<br>>70: 48.8% | Cancer registry Munich [35] |
| Survival by T-category 1-year | T1: 93.8%<br>T2: 85.3%<br>T3: 79.9%<br>T4: 76.2% | T1: 95.8%<br>T2: 90.6% (*)<br>T3: 81.7%<br>T4: 71.7% | T1: 96%<br>T2: 93%<br>T3: 72%<br>T4: 71% | Cancer registry North Rhine Westphalia [34] |
| 5-year | T1: 73.3%<br>T2: 55.8%<br>T3: 46.9%<br>T4: 35.2% | T1: 75.8%<br>T2: 62.3% (*)<br>T3: 50.6%<br>T4: 36.2% | T1: 86%<br>T2: 65%<br>T3: 42%<br>T4: 40% | Cancer registry North Rhine Westphalia [34] |
| Survival by UICC 1-year | I: 94.9%<br>II: 89.6%<br>III: 85.9%<br>IV: 73.9% | I: 95.6%<br>II: 93.4%<br>III: 87.4%<br>IV: 73.5% | Not available | Not available |
| 5 years | I: 75.6%<br>II: 63.2%<br>III: 56.1%<br>IV: 33.7% | I: 77.5%<br>II: 69.1%<br>III: 60.1%<br>IV: 37.3% | Men/Women -> Mean value<br>I: 84/86 -> 85<br>II: 72/64 -> 68<br>III: 50/53 -> 52<br>IV: 40/39 -> 40 | ZFKD [18] |

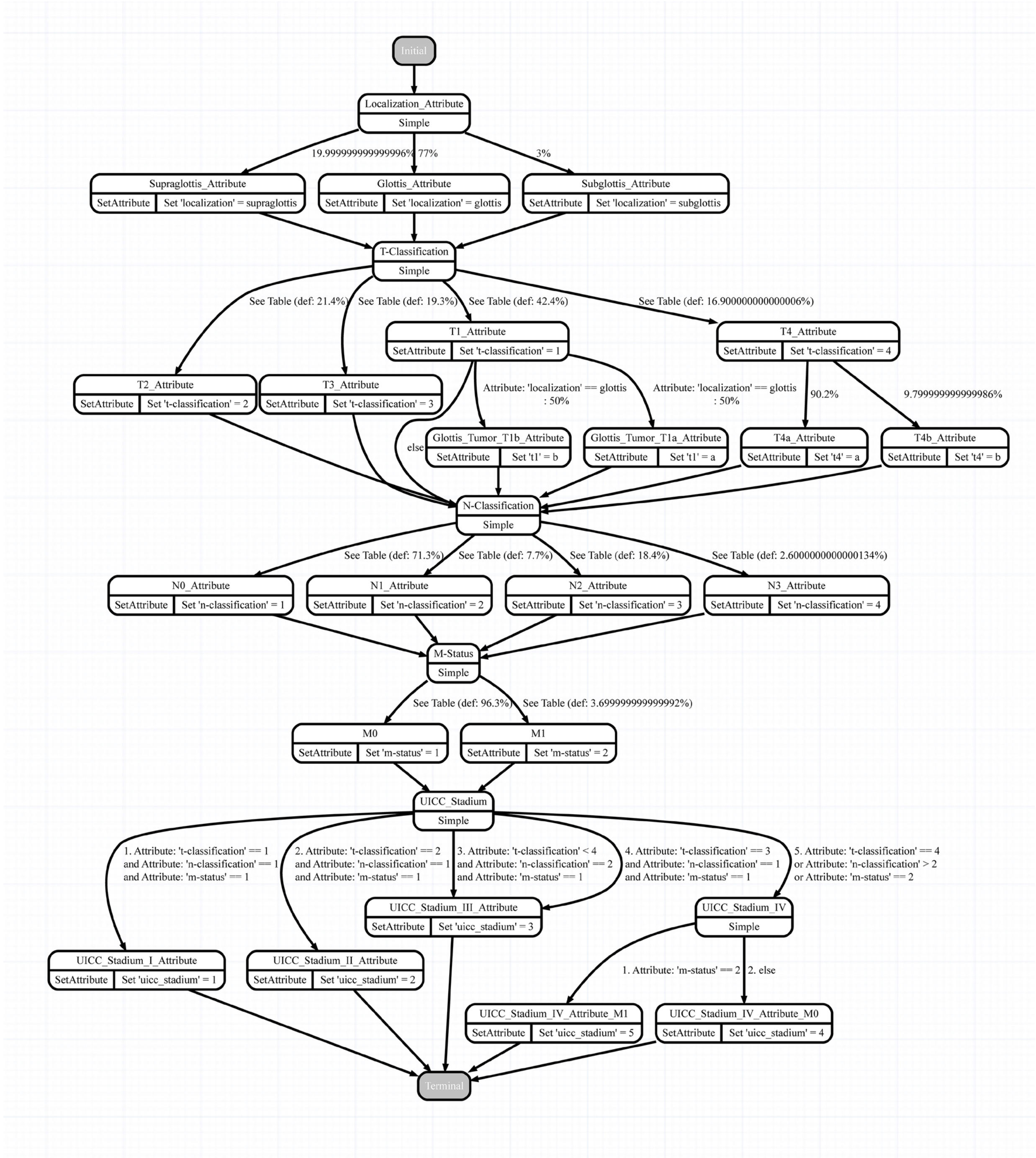


**Figure S1.** "tnm_distribution_localization_first_diagnosis" submodule displayed in the Synthea Generic Module Builder

**Description Figure S1.**

In this submodule, the tumor characteristics of the laryngeal carcinoma are defined for the patients that suffer from laryngeal cancer. The logic for a single patient running through the submodule, the used states and the used transitions are explained below. A more detailed list and explanation of all states and transitions can be found here: “Generic Module Framework” chapter of Synthea's wiki on their GitHub page (https://github.com/synthetichealth/synthea/wiki/Generic-Module-Framework). The "Initial" state represents the starting point of the module at which the patient "enters" the module. From this, a transition of the "direct" type leads to the next state. The direct transition leads the patient to the subsequent state. In this case, the next state is a "simple" state that has no direct influence on

the simulation and will not appear in the record. It serves the programmer for a better overview and, if necessary, to merge previously split paths. From the “Localization_Attribute”-state onwards, the previously stringent path is divided into three paths by a "distributed" transition. In this transition, a frequency is defined that determines how likely it is that the patient will be "sent" to the downstream states. Here, this means that our patient is sent to the “Glottis_Attribute”-state with a probability (p) of p = 0.77, to the “Supraglottis_Attribute”-state with p = 0.2 and to the “Subglottis_Attribute”-state with p = 0.03. In the subsequent “SetAttribute” state, the tumour localization is stored in a variable that can be accessed across modules. If we now briefly consider this association in the context of a larger population, it means that 77% of patients get a carcinoma in the glottis, 20% in the supraglottis and 3% in the subglottis. However, since Synthea determines the variables independently for each patient, these p-values correspond more to an expected value and little deviations may occur, especially in small samples. Next, all three paths are merged back into one and the patient arrives at the “T-Classifications” state, regardless of which attribute he or she previously received. From there, the patient can reach 1 of 4 possible states, that choose the T-Stage, through a table transition. With this type of transition, certain cases are predefined in a CSV-table under which specific p-values are then used. For example in this case: if gender = male, then p(T1_Attribute) = 0.43, p(T2_Attribute) = 0.21, p(T3_Attribute) = 0.18, p(T4_Attribute) = 0.17. Any number of conditions can be added, which are then linked by the logical operator “AND”. The N-stage and M-stage are assigned to the patient according to the same principle. The patient is then assigned a suitable UICC. This can be achieved using a conditional transition. Typical logical operators are available here: if, else, and, or. For the patient, this means for example that if he or she has been assigned a T1 stage, N0 stage and M0 stage, he will reach the “UICC_Stage_I_Attribute”-state and thus be assigned UICC I. Finally, the patient reaches the “Terminal” state, which represents the end of the module, and leaves the module.
